# Use and acceptance of long lasting insecticidal net screens for dengue prevention in Acapulco, Guerrero, Mexico

**DOI:** 10.1186/1471-2458-14-846

**Published:** 2014-08-14

**Authors:** Catrin H Jones, David Benítez-Valladares, Guillermo Guillermo-May, Felipe Dzul-Manzanilla, Azael Che-Mendoza, Mario Barrera-Pérez, Celia Selem-Salas, Juan Chablé-Santos, Johannes Sommerfeld, Axel Kroeger, Timothy O’Dempsey, Anuar Medina-Barreiro, Pablo Manrique-Saide

**Affiliations:** Liverpool School of Tropical Medicine, Pembroke Place, Liverpool, L3 5QA UK; Special Programme for Research and Training in Tropical Diseases, World Health Organization, (TDR, WHO), Av Appia, Geneva, 1211 Switzerland; Universidad Autónoma de Yucatán, Carretera Mérida-Xmatkuil Km. 15.5, Mérida, C.P. 97315 Mexico; Servicios Estatales de Salud de Guerrero, Avenue Ruffo Figueroa No. 6, Col. Burócratas, Chilpancingo, C. P. 39090 México; Servicios de Salud de Yucatán, Laboratorio Estatal de Salud Pública, C. 39-C No. 345-A X 2-A Y 4 Col. Mayapán, Merida, C.P. 97159 México

**Keywords:** Dengue vectors, Long lasting insecticidal nets, Door and window screens, Community acceptance, Mexico

## Abstract

**Background:**

Dengue, recognized by the WHO as the most important mosquito-borne viral disease in the world, is a growing problem. Currently, the only effective way of preventing dengue is vector control. Standard methods have shown limited effect, and there have been calls to develop new integrated vector management approaches. One novel tool, protecting houses with long lasting insecticidal screens on doors and windows, is being trialled in a cluster randomised controlled trial by a joint UADY/WHO TDR/IDRC study in various districts of Acapulco, Mexico, with exceptionally high levels of crime and insecurity.

This study investigated the community’s perspectives of long lasting insecticidal screens on doors and windows in homes and in schools, in order to ascertain their acceptability, to identify challenges to further implementation and opportunities for future improvements.

**Methods:**

This was a sequential mixed-methods study. The quantitative arm contained a satisfaction survey administered to 288 houses that had received the intervention examining their perspectives of both the intervention and dengue prevention in general. The qualitative arm consisted of Focus Group Discussions (FGDs) with those who had accepted the intervention and key informant interviews with: schoolteachers to discuss the use of the screens in schools, program staff, and community members who had refused the intervention.

**Results:**

Overall satisfaction and acceptance of the screens was very high, with only some operational and technical complaints relating to screen fragility and the installation process. However, the wider social context of urban violence and insecurity was a major barrier to screen acceptance. Lack of information dissemination and community collaboration were identified as project weaknesses.

**Conclusions:**

The screens are widely accepted by the population, but the project implementation could be improved by reassuring the community of its legitimacy in the context of insecurity. More community engagement and better information sharing structures are needed.

The screens could be a major new dengue prevention tool suitable for widespread use, if further research supports their entomological and epidemiological effectiveness and their acceptability in different social and environmental contexts. Further research is needed looking at the impact of insecurity of dengue prevention programmes.

**Electronic supplementary material:**

The online version of this article (doi:10.1186/1471-2458-14-846) contains supplementary material, which is available to authorized users.

## Background

The incidence of dengue is rapidly growing, with a 30-fold increase over the past 50 years [1]. Dengue is recognised as a public health emergency of international concern [2] and the most important mosquito-borne viral disease in the world [1]. Today, 2.5 billion people (40% of the world’s population) live in areas at risk of dengue [3]. Dengue disproportionately affects poorer people [1], and can strongly affect a vulnerable household’s economic security.

The only established method of dengue prevention is vector control [4]. The effectiveness of “traditional” methods, such as episodic insecticide space-spraying [5] and top-down vertical programmes [6] has been questioned. The development of innovative integrated vector management (IVM) programs with novel instruments and approaches is a priority [3], with greater awareness of the sociocultural needs and participation of affected communities in vector control [7]. Program sustainability is key, as mosquitoes rapidly return if prevention methods are reduced or stopped [8].

Morrison et al. [4] argue that a major factor in the failure of previous prevention methods is their focus on eliminating immature forms of *Ae. aegypti,* rather than target the adult mosquitoes that actually transmit the disease. Recently, the use of long-lasting insecticidal nets (LLINs) has been proposed as a possible intervention. The rationale is that the LLINs stop human-vector contact by physically blocking the entry of mosquitoes and the insecticide reduces the mosquito population able to transmit dengue by either killing them or reducing their life expectancy [9]. The fabric retains its efficacy for at least one year [10], and minimal behaviour change is needed from the recipient. Theoretically, LLINs could be a simple, effective tool in the IVM arsenal [11].

Kroeger et al. [12] and Lenhart et al. [13] both found that LLINs deployed on doors and windows as curtains combined with targeted treatment of breeding sites reduced dengue vector levels and could interrupt transmission. However, Vanlerberghe et al. [14] found that coverage of the LLIN curtains fell over time. Rizzo et al. [15] also found this, noting that families would tie back the curtains to increase ventilation during the day, compromising the utility of the intervention. A possible solution to this is to permanently fix the LLIN to the doors and windows in the form of a screen; this is the intervention being trialled in Acapulco.

The use of LLINs for dengue have been shown to be well accepted by recipient communities [12, 15]; however Kroeger et al. [16] found that fear of insecticides, lack of knowledge, perceived lack of need and little incentive for community participation impeded the demand for similar LLINs for use against malaria in Mexico. Acapulco is the first centre to use screens rather than curtains, so little is known about their effectiveness at preventing dengue or community perspectives and acceptance of them in homes and public buildings.

The World Health Organization Special Programme for Research and Training in Tropical Diseases (WHO TDR) and International Development Research Centre (IDRC) have launched two multi-centre trials, one in South-East Asia [17] and one in Latin America [18], studying the impact of an eco-bio-social approach to vector management. This study was undertaken as part of one arm of the Latin American multi-centre trial with a cluster-randomised sampling design with cross-sectional entomological surveys to assess efficacy [12]. Duranet^®^ screens (0.55% w.w. alpha-cypermethrin-treated non-flammable polyethylene netting [145 denier; mesh = 132 holes/sq. inch]; Clarke Mosquito Control, IL, USA; WHOPES approved) were mounted in aluminum frames custom-fitted to doors and windows of residential houses. Project staff installed the screens using either screws or plastic ties depending on the house structure and householder preference. The installation, in collaboration with a local small business from the locality and the Ministry of Health (MoH), started in April 2012. In total, 746 households received the intervention.

This study aims to describe the community’s baseline knowledge, understanding and attitudes towards dengue and their current prevention practices; explore the acceptance, use, adherence, and perspectives of the long-lasting insecticidal screens; and offer suggestions about how to alter the programme to better address the socio-cultural needs of the community.

## Methods

### Study site

The study took place in Acapulco de Juarez, a coastal city of 750,000 people located in the state of Guerrero on the Pacific coast of Mexico. The local economy is heavily dependent on tourism: annually, 4.5 million tourists visit Acapulco bay, and 72.92% of Acapulco’s inhabitants are engaged in tourism-based economic activity [19]. The neighbourhood of Ciudad Renacimiento is a primarily residential area located in the north of Acapulco. It has 11,725 premises and 48,460 inhabitants (6.55% of total population of Acapulco) [20].

Mexico, with an average of 75,355 annual cases, had the fourth highest average number of dengue cases in the world in 2004–2010 [3], and the state of Guerrero had the third-highest incidence in Mexico [21]. Acapulco is a dengue hot spot within Guerrero, consistently reporting more than 30%-50% of all annual cases [22]. The Ministry of Health (MoH) recognises Renacimiento as a high-risk area within Acapulco.

As an impoverished urban area in a major international tourist destination, Renacimiento displays most of the risk factors for dengue. It is poorly served by public amenities: water supply and waste collection are irregular and badly organised, resulting in water storage and waste accumulation, which provides ideal conditions for breeding sites. Sixty to seventy per cent of its houses have open walls and unprotected windows, allowing intradomicilliary mosquito-human contact (P. Manrique-Saide personal communication).

Mexico has seen a dramatic rise in violence since President Felipe Calderon declared a ‘War on Drugs’ in 2006, and initiated a deeply militarised approach to counter-narcotics. Insecurity has seeped into all aspects of life in Acapulco: in 2012, there were 2,754 homicides, 351 rapes, 75 kidnappings and 15,135 reported robberies in Guerrero [23]. These circumstances had a major impact on the study, as explained below.

### Study design

This study employed a sequential mixed method design with a quantitative and a qualitative arm, in order to produce generalizable, categorical data describing the community’s practices on dengue prevention and satisfaction regarding the installation of mosquito screens by the intervention, and to explore and contextualise different perspectives held within the community.

### Quantitative data

The quantitative arm of this research was a cross-sectional descriptive study containing a multiple-choice satisfaction survey together with a small screen condition survey. The satisfaction survey focused in great detail on general dengue prevention practices, attitudes to dengue prevention, use and effect of the mosquito screens, and satisfaction with the mosquito screen project (Additional file 1). This survey was carried during May and June 2013.

### Qualitative data

Grounded theory was chosen as the theoretical underpinning of the research strategy. The qualitative data collection was conducted during the months of June and September of 2013. Focus group discussions (FGDs) were used to explore the views of those who had accepted the screens, aiming to capture a range of perspectives and identify common views and experiences amongst recipients.

Semi-structured interviews were conducted with 3 groups of key informants: school teachers, program staff, and people who refused the screens (Table 1). This methodology was chosen for the schoolteachers and the program staff as their role (in school-based prevention methods, and project implementation respectively) meant that they were uniquely positioned to have key information on particular aspects of the project, and therefore a one-on-one interview focusing on capturing their deep knowledge was more appropriate than a group-based methodology [24].

**Table 1:** **Sampling matrix for the qualitative arm of the study of use and acceptance of long lasting insecticidal screens on doors and windows in houses and schools for dengue prevention in Acapulco, Guerrero, Mexico**

Method	Number recruited	Recruitment criteria
Focus groups	6 groups:	Accepted the mosquito screens
	FGD1 ➔ 5 participants (women)	
	FGD2 ➔ 6 participants (men)	
	FGD3 ➔ 3 participants (men)	
	FGD4 ➔ ^▪^ 8 participants (women)	
	FGD5 ➔ ^▪^ 10 participants (men)	
	FGD6 ➔ 8 participants (women)	
Key Informant Interviews with school teachers	3 semi-structured interviews	Responsible for the screens in the school
Key Informant Interviews with project staff	3 semi-structured interviews	Key role in project implementation
Key Informant Interviews with people who rejected	2 semi-structured interviews	Rejected the mosquito screens

One-on-one interviews were used with people who refused the project because they were a very difficult population to recruit, and interviews are more accessible than focus groups [25].

A semi-structured format was chosen because the loose structure allowed comparable data to be collected between different key informants in the same group, whilst maintaining sufficient flexibility to react and further probe arising issues [24].

### Sampling strategy for quantitative data

The sampling frame was 10,711 households in the suburbs of Renacimiento and Zapata. The area was divided using satellite imaging into clusters of approximately 100 houses. Twenty clusters were randomly selected, resulting in a study population of 2000 households. Ten clusters were randomly selected to receive the intervention and 10 to be controls. One thousand households were eligible to receive the intervention, of which 746 households accepted and 254 rejected it.

Ideally, all 746 intervention households would be surveyed in the satisfaction survey. However, in light of the current increase of violence and the subsequent reluctance to participate in the study in Renacimiento, it was decided that this was unrealistic. Therefore a quota of 373, half of the households, was selected as the sample size.

A quota design is not random and is vulnerable to selection bias, but it is justifiable in this case as a pragmatic compromise. As Deanscombe states [24], non-probability sampling can allow sufficiently representative samples to be collected within the time and contextual constraints. Most data collection occurred during weekdays, but weekend and evening data collection were conducted to reduce selection bias, and efforts were made to visit every cluster multiple times.

### Sampling strategy for qualitative data

Sampling was purposive, aiming to capture a wide range of perspectives. The principle of saturation guided the sample size, detailed in Table 1.

The LSTM Masters Ethics Committee approved this study, and the Internal Review Board of UADY and the Ethical Review Committee of the WHO approved the larger UADY study, including this work.

Informed, voluntary consent was obtained from all survey, focus group and interview participants.

### Data analysis

#### Quantitative data

The satisfaction survey was entered into SPSS 20, with 10% double inputted by a second researcher to check for errors. The written responses were collected and tabulated using Microsoft Word. Common unanticipated responses for ‘others’ and suggestions for improvement were retrospectively coded.

SPSS 20 was used to calculate frequency counts, and to create contingency tables to compare the association between variables. The statistical significance of this relationship was tested using the Fisher exact test. Stata 9.2 was used to calculate cluster-adjusted confidence intervals (CIs) at a confidence level of 95%.

### Qualitative data

Data were analysed using a grounded theory approach. This process was carried out through different stages: a) familiarisation, with detailed readings of the transcripts; b) identifying and reaching consensus by the researchers group of a thematic framework from the transcripts; c) indexing and charting using Nvivo 9 for coding and data management; and d) mapping and interpretation of the data.

The data from the quantitative surveys were added to the charted qualitative data, and compared with the emerging themes and concepts generated by the qualitative analysis process. The mixed-method nature of this study allows for triangulation between data collected in the satisfaction survey, focus groups and interviews. Comparing and converging different sources of data allows a more complete picture to be built as each offer a different insight on the research problem [26], and increases accuracy by allowing confirmation of the findings [24].

## Results

Two hundred and eighty-eight surveys were collected (77% of the quota). The population sampled for the satisfaction survey was disproportionally female (75.3%), with a wide range of ages (18-87years). A possible explanation could be that men are away working during most of the day. The most common occupation was housewife (63.5% of all participants, 83.8% of female participants).

### Perspectives of prevention practices

Data collected in both arms of the study strongly suggest that many in Renacimiento are actively taking preventative measures against dengue. Most of the respondents (78.1%) in the satisfaction survey reported that they destroyed breeding sites in their homes. The main reasons given for not taking measures to destroy breeding sites were that the respondents were too busy, or that they perceived it to be unnecessary.

In the satisfaction survey, 15.6% reported that they participated in collective community activities to prevent dengue. The most common activities were participation in clean-up campaigns, destruction of breeding sites and information sharing.

Fumigation (space spraying) was perceived to be the most effective method of preventing dengue, with 40.3% of respondents to the satisfaction survey identifying it as such.

A strong feeling of individual responsibility for dengue prevention was evident. This was reflected in the FGDs, where individual households were perceived to hold primary responsibility for preventing dengue by maintaining high hygiene standards and educating their children: “Cleaning every day, cleanliness everywhere, the house being clean is the main thing for there not being any flies, so the mosquitoes don’t slip in” *Participant 1, FGD4.*

Moreover, 82.3% of survey respondents selected individuals as having the greatest responsibility for prevention. Governmental action, though regarded as beneficial and desirable, was seen as secondary to the individual’s responsibility to protect their family.

Overall satisfaction with governmental dengue prevention efforts was high in the quantitative and qualitative arms.

### Perspectives about the screens

#### Recruitment process

The recruitment process was not well understood by the community. Many participants from the FGDs did not know why they had been offered the screens while others had not, and this had placed some in difficult situations with neighbours and family who wanted to know why the participant had received seemingly preferential treatment.

Many focus groups participants identified information giving as a weak point within the recruitment process. Several reported only being told that the nets would stop mosquitoes and protect them against dengue, with some only discovering that the net contained insecticide while participating in the focus groups. Others reported that they were unsure how to clean and care for them.

### Reasons for acceptance

The main reason for acceptance was that the screens were perceived to be beneficial. However, opinions about the screens were not the main factor considered when people decided whether to accept the project. The crucial factor was often perceived, by the FGDs participants, to be whether, in light of the current security situation, they felt that they trust those promoting and implementing the process. Clear identification as health workers was seen as a vital part of gaining trust, and therefore participation, in the project.

Project staff reported that the initial houses were very difficult to recruit, but once a few houses had been completed in an area and news of the project had spread, more people accepted. Hearing and being able to see that the project was legitimate sufficiently reassured people.

### Reasons for rejection

The fear of violence was frequently identified as a common reason for the high rejection rate (around 25% of households):

“What is missing is that they [the local people] do not trust [anything] because here in the 10th [block] there was one woman alone, because of course they had already killed one of her sons, they went to her house, they came for malaria but they entered, robbed her and killed her (…) [the robbers] said that they came from the health centre, but they were deceitful.” *Participant 6, FGD2.*

“As various people had died, well… been killed more than anything, in that same cluster, people didn’t go out, they didn’t even poke their heads out to see it was true or not, they said- no, no, no. And then there was rejection” *Project Staff 1.*

Though only one group reported murder by public health worker impersonators, other focus groups and key informants reported that thieves impersonated public health workers in order to gain access into homes, suggesting that this is a commonly held belief within the community and a major contributor to rejection.

Another factor that was perceived to have increased rejection was a rumour that the screens were not actually free, and that once the screens were installed there would be a charge.

Though a minor factor compared to the effect of insecurity on acceptance, project staff and participants in one focus group believed that misgivings about the screens, particularly fears that they would reduce air circulation and therefore increase room temperature, caused some rejection. Lack of awareness and understanding of the project within the community was also identified as reasons for rejection and avoiding recruitment.

### Installation

In the satisfaction survey 94.4% reported that they were happy with the way that they had received their screens (Additional file 2). However, during the FGDs, some problems were unearthed.

A total of 50.3% of the households had screens on every window and door, and 49% had screens on some of the windows and doors. This high level of partial or incomplete installation is reflected in the experiences of many of the focus group participants, and this partial installation was the most common grievance with the project.

Some incomplete installations resulted from participants’ refusal and structural difficulties installing screens, especially in houses constructed with an open front. However, most participants in the focus groups who complained about the incomplete installation were both willing and suitable candidates for screen installation but the work had not been completed because of operational challenges.

### Benefits of the screens

**Figure 1:**
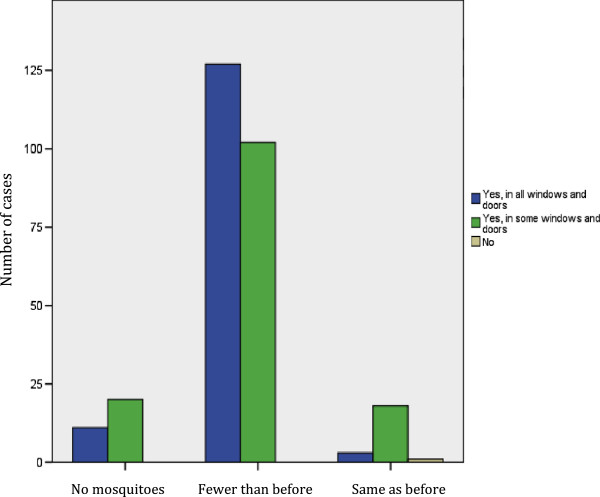
**Comparison of reported change in domestic mosquito numbers with the installation status of long lasting insecticidal screens on doors and windows in Acapulco, Guerrero, Mexico.**

The most notable benefit reported for the screens were the effect they had on the amount of mosquitoes in the home. Following screen installation, 79.9% of recipients reported that there were fewer mosquitoes in the home and a further 10.8% reported that there were none: only 7.6% reported that the amount was the same as before (Figure 1, Additional file 2). Those who had only had a partial installation were significantly more likely (p < 0.001) to report no change in the amount of mosquitoes in the home.

The reduction in the amount of mosquitoes in the home was associated with a reduction in mosquito biting in the home: 88.5% or recipients reported that mosquito biting was less of a nuisance within their homes after receiving the screens (Additional file 2). Again, those who only had partial installation were significantly more likely to report no change in mosquito biting (p = 0.02).

FGD participants also appreciated a reduction in mosquito numbers. Though many were unaware that the net contained insecticide, they had noticed that mosquitoes and other pests died on contact with the screen, and were satisfied with this.

The insecticide in the screen was seen as beneficial and acceptable, with few reports of side effects or fears about its use.

The quantitative and qualitative data both suggest that other insect pests were similarly reduced. Focus group participants reported a reduction in flies and cockroaches, and 79.9% of satisfaction survey participants reported a reduction in other pests. The majority of participants (90.2%) had the same amount or more screens in place now compared to the original amount installed, suggesting that very few were removed.

### Problems with the screens

The main problem identified with the screens once installed was fragility, especially door screens and in schools. The satisfaction survey found that the most common reason for a house to have fewer screens now than originally installed was screen breakage (44%), and higher quality material was the most frequently suggested improvement for the project.

**Figure 2:**
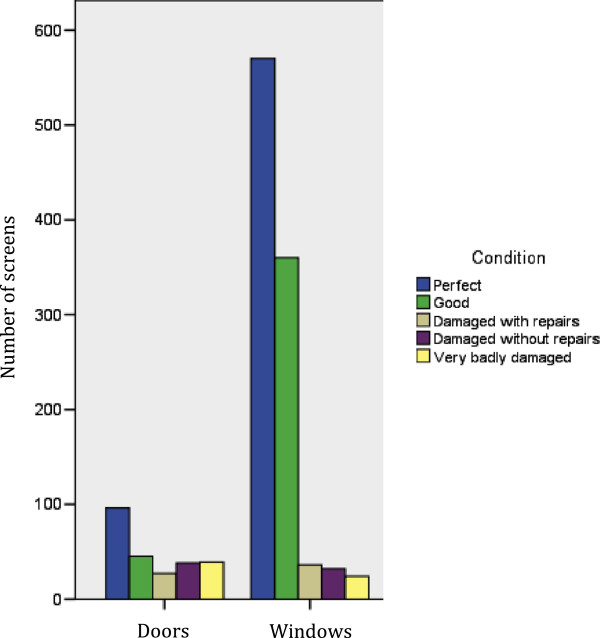
**Comparison of the condition of doors and window screens.**

A survey of the screens found that the windows were broadly in very good condition, while the doors were faring less well, with 42.4% damaged in some way (Figure 2).

Screen fragility was also reported in the FGDs. Some screens were broken in exceptional accidents, but the majority of breakages reported in the focus groups occurred during normal use.

Though some participants had feared a reduction in air circulation prior to installation, none had experienced this problem. Indeed, many expressed surprise that they had felt no effect.

Overall satisfaction with the project was very high. 80.9% scored their satisfaction with the screens as 5/5, and 89.9% gave a score of either 4 or 5 (Additional file 2). 99.3% would recommend the project to another city (Additional file 2). This was reflected in the FGDs, where participants would often praise the project and qualify any criticism with a caveat that they were still grateful for the screens overall.

Contrary to experiences in homes, all three school key informants reported that the screens had reduced air circulation and increased classroom temperatures, which is perhaps understandable considering that classrooms have a far higher occupancy during the hottest time of the day compared with homes, so even minor changes in air circulation could have a noticeable effect.

### Suggested improvements

**Figure 3:**
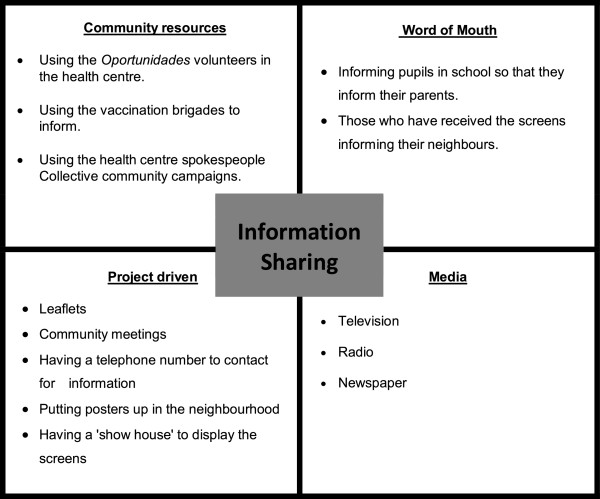
**Suggested methods of improving the information sharing process.**

Because of the mistrust and insecurity, reliable and proactive information sharing about the project was seen as key to establishing trust, and was widely highlighted as an area for improvement. FGDs participants suggested a variety of information sharing methods that the project could use to improve communication (Figure 3).

Many FGD participants and the schoolteachers were enthusiastic to work closer and collaboratively with the project, and many saw this as a way of increasing community acceptance. Schoolteachers expressed a desire to take a proactive role in informing parents of the nets. Active collaboration with agents within the community such as the health centres that the community trust was also seen as a way of improving acceptance.

## Discussion

### Screens as a dengue prevention tool

As far as we are aware, no study has been published previously specifically concerning insecticidal screens for the prevention of dengue. All aspects of the data in this study suggest that the screens were widely accepted. The screens comply with McCall and Kittayapong’s criteria [17] for a good prevention tool: they were seen as user-friendly and desirable, and required negligible behaviour change. The high levels of satisfaction with ITMs are similar to the findings of Lenhart et al. [13] and Kroeger et al. [12] with little evidence of the fears concerning insecticide described by Kroeger et al. [16] for insecticide treated bed nets.

This study indicates that in terms of desirability screens compare favourably to insecticide treated curtains. Contrary to what Rizzo et al. [15] and Vanlerbergher et al. [14] found with curtains, this study found that only a very small proportion of households had fewer screens than originally installed, suggesting that they are rarely intentionally removed. Additionally, there were fewer complaints about appearance than indicated by Rizzo et al. [15]. The fragility of the screen material is a necessary compromise between allowing the passage of air and light and maintaining sufficient robustness to withstand normal wear and tear, nevertheless there is room for improvement in screen maintenance and repair, and possibly in the design of door frames.

However, the screens do not address dengue’s causative factors or the wider social context. The prevalence of dengue and the prevalence of insecurity are not completely independent phenomena affecting the same community. Insecurity is an immense barrier to effective dengue prevention, and insecurity and dengue share many common causal and facilitative factors deeply rooted in inequality, poverty, inadequate public service provision, poor housing and lack of opportunities. These factors facilitate the flourishing of the informal criminal economy, encourage the spread of *Ae. aegypti* breeding sites and peoples’ vulnerability to bites in overcrowded poorly-built homes, and impede any effort to deal with either problem.

## Conclusions

Key measures in implementing successful programmes in insecure urban settings that have been developed in the field of humanitarian action could be applied to future dengue prevention programmes. A thorough situational analysis to recognise and accommodate the challenges posed by insecurity [27, 28], meaningful community engagement with good communication and formation of partnerships and networks with existing civil society groups and the local authority [29, 27, 30]; and a robust monitoring and evaluation structure to react to rapid changes in the situational dynamics and collect feedback [31] could all help to surmount the barriers posed by insecurity.

Two key areas were identified where the project could improve on their engagement:

1. **Directly with individuals:** collaboration could be improved by engaging with individual households, actively seeking their feedback at all stages of implementation, learning from complaints and reflexively adapting the program in line with the responses. This is in line with Inter-agency Standing Committee (IASC) guidelines for ensuring programmatic accountability to recipients [32].
2. **With existing civil society groups (CSG):** Strong, networked social capital that is both locally independent and legitimate in the eyes of the community, has been identified as instrumental to the success of community-based programmes [33]. By engaging and collaborating with existing CSGs already legitimate in the eyes of the community, such as church groups, parent groups at schools and the local *‘Oportunidades’* volunteer health brigades, the project could utilise their social capital to increase its own legitimacy and reach. Collaboration with CSGs could also increase community ownership of the project, and assist with information distribution as described below. A recommendation is offered that the project actively reaches out to different CSGs to discuss with them possible ways that they could collaborate with the project.

As outlined above, communication has been a weak point for the project. Two improvements are recommended:

1. **Collaboration with Mexican authorities:** an information campaign to inform the whole community about the project was widely called for by the FGD participants. The project could collaborate with the MoH at municipal, state and federal level to inform the community by integrating information about the project into MoH campaigns. Other ministries that should be involved in order to improve or to scale up this project are the Ministry of Social Development (SEDESOL) and the Ministry of Education (SEP).
2. **Follow-up and support:** FGD participants reported that they were often ill informed about the practicalities of the installation process and screen maintenance. A recommendation is offered that the project has a phone number, text service and/or drop-in centre that participants can use.

### Limitations of the study

The most significant limitation is the sampling for the satisfaction survey. Nonparticipation was high, and only 77% of the quota amount was met (39% of the total amount of households with screens). There is evidence of a selection bias in the quantitative arm despite the efforts taken to minimise it: females are overrepresented and younger people, those who work away from home, and males are all underrepresented.

## Electronic supplementary material

Additional file 1: **Questionnaire key concepts and indicators.** (DOC 37 KB)

Additional file 2: **Summary of the results of the satisfaction survey on the use and acceptance of long lasting insecticidal screens on doors and windows for dengue prevention in Acapulco, Guerrero, Mexico.** (DOC 474 KB)

## References

[1] World Health Organization (2009). Dengue: Guidelines for Diagnosis, Treatment, Prevention and Control.

[2] World Health Organization: **Revision of the International Health Regulations.World Health Assembly resolution WHA 58.3, adopted by the 58th World Health Assembly**. http://www.who.int/csr/ihr/WHA58-en.pdf

[3] World Health Organization (2012). Global Strategy for Dengue Prevention and Control 2012–2020.

[4] Morrison AC, Zielinski-Gutierrez E, Scott TW, Rosenberg R (2008). Defining challenges and proposing solutions for control of the virus vector aedes aegypti. PLoS Med.

[5] Esu E, Lenhart A, Smith L, Horstick O (2010). Effectiveness of peridomestic space spraying with insecticide on dengue transmission; a systematic review. Trop Med Int Health.

[6] Toledo ME, Baly A, Vanlerbergue V, Rodrieguez M, Benitez JR, Duvergel J, Van Der Stuyft P (2008). The unbearable lightness of technocratic efforts at dengue control. Trop Med Int Health.

[7] Parks W, Lloyd L: **Planning social mobilization and communication for dengue fever prevention and control. a step-by-step guide**. http://www.who.int/tdr/publications/documents/planning_dengue.pdf

[8] Nathan M (2012). Introduction. Pathog Glob Health.

[9] Vanlerberghe V, Villegas E, Oviedo M, Baly A, Lenhart A, McCall PJ, Van der Stuyft P (2011). Evaluation of the effectiveness of insecticide treated materials for household level dengue vector control. PLoS Negl Trop Dis.

[10] Vanlerberghe V, Trongtokit Y, Cremonini L, Jirarojwatana S, Apiwathnasorn C, Van der Stuyft P (2010). Residual insecticidal activity of long-lasting deltamethrin treated curtains after 1 year of household use for dengue control. Trop Med Int Health.

[11] Loroño-Pino MA, García-Rejón JE, Machain-Williams C, Gomez-Carro S, Nuñez-Ayala G, del Nájera-Vázquez M R, Losoya A, Aguilar L, Saavedra-Rodriguez K, Lozano-Fuentes S, Beaty MK, Black WC, Keefe TJ, Eisen L, Beaty BJ (2013). Towards a casa segura: a consumer product study of the effect of insecticide- treated curtains on aedes aegypti and dengue virus infections in the home. Am J Trop Med Hyg.

[12] Kroeger A, Lenhart A, Ochoa M, Villegas E, Levy M, Alexander N, McCall P J (2006). Effective control of dengue vectors with curtains and water container covers treated with insecticide in Mexico and Venezuela: cluster randomized trial. Br Med J.

[13] Lenhart A, Orelus N, Maskill R, Alexander N, Streit T, McCall PJ (2008). Insecticide-treated bednets to control dengue vectors: preliminary evidence from a controlled trial in Haiti. Trop Med Int Health.

[14] Vanlerberghe V, Villegas E, Jirarojwatana S, Santana N, Trongtorkit Y, Jirarojwatana R, Srisupap W, Lefèvre P, Van der Stuyft P (2011). Determinants of uptake, short term and continued use of insecticide-treated curtains and jar covers for dengue control. Trop Med Int Health.

[15] Rizzo N, Gramajo R, Cabrera-Escobar M, Arana B, Kroeger A, Manrique-Saide P, Petzold M (2012). Dengue vector management using insecticide treated materials and targetted interventions on productive breeding-sites in Guatemala. BMC Public Heath.

[16] Kroeger A, Avinna A, Ordonez-Gonzalez J, Escandon C (2002). Community cooperatives and insecticide-treated materials for malaria control: a new experience in Latin America. Malar J.

[17] McCall PJ, Kittayapong P, WHO (2007). Control of Dengue Vectors: Tools and Strategies. Report of the Scientific Working Group Meeting on Dengue, Geneva, 15 October 2006.

[18] World Health Organization TDR: **Nine projects to find new solutions to dengue and Chagas disease: An eco-bio-social approach in Latin America and the Caribbean**. http://www.who.int/tdr/news/2011/dengue-chagas-new-solutions/en/index.html

[19] Espinosa Medina O (2004). La medición de las capacidades y la dedicación de los empleados en el sector hotelero de Acapulco, Guerrero.

[20] ***Conteo de Poblacion y Vivienda*****(Population and Housing census)**. http://www.inegi.org.mx/est/contenidos/proyectos/ccpv/cpv2005/Default.aspx

[21] Fajardo-Dolci G, Meljem-Moctezuma J, Vicente-González E, Vicente Venegas-Páez F, Mazón-González B, Gerardo Aguirre-Gas H (2012). El dengue en Mexico: conocer para mejorar la calidad de la atención (Dengue in Mexico: recognise in order to improve the quality of the attention). Rev Inst Mex Seguro Soc.

[22] Secretary of Health: *Sistema Nacional de Vigilancia Epidemiologica (National System of Epidemiological Surveillance)*. Mexico; http://www.rhove.gob.mx/

[23] Chouza P: **Acapulco: sol, playa y violencia (Acapulco: sun, beach and violence;)**. http://internacional.elpais.com/internacional/2013/03/23/actualidad/1364073762_285806.html

[24] Deanscombe M (2010). The Good Research Guide for Small Scale Research Projects.

[25] Lewis J, Richie J, Lewis J (2003). Design Issues. Qualitative Research Practice: A Guide for Social Science Students and Researchers.

[26] Mays N, Pope C (2000). Assessing quality in qualitative research. Br Med J.

[27] Lucchi E (2010). Between war and peace: humanitarian assistance in violent urban settings. Disasters.

[28] Lucchi E (2012). Moving from the ‘why’ to the ‘how’: reflections on humanitarian response in urban settings. Disasters.

[29] Sanderson D, Knox Clarke P, Campbell L: **Responding to Urban Disasters: Learning from previous relief and recovery operations**. http://www.alnap.org/resource/7772.aspx

[30] **Operational security management in violent environments**. http://www.odihpn.org/index.php?option=com_k2&view=item&layout=item&id=3159

[31] Koscalova A: **Needs Assessment. Discussion papers 22: Humanitarian Interventions in Urban Settings**. https://www.msf.es/sites/default/files/publicacion/cuaderno-22.pdf

[32] Accountability to Affected Populations: **Tools to assist in implementing the IASC AAP commitments**. http://www.fao.org/emergencies/resources/documents/resources-detail/en/c/175109/

[33] Pelling M (2003). The Vulnerability of Cities: Natural Disasters and Social Resilience.

[CR34] The pre-publication history for this paper can be accessed here: http://www.biomedcentral.com/1471-2458/14/846/prepub

