# Supplementary material for: Use and acceptance of long lasting insecticidal net screens for dengue prevention in Acapulco, Guerrero, Mexico
Source: BMC Public Health. 2014 Aug 14;14:846. doi: 10.1186/1471-2458-14-846 (PMC4152567; doi:10.1186/1471-2458-14-846)
Supplement: Supplementary file 1 — Additional file 1: Questionnaire key concepts and indicators. (DOC 37 KB) [file 12889_2014_6989_MOESM1_ESM.doc]

**Additional file 1** Questionnaire key concepts and indicators

| Key concepts | Indicators |
| --- | --- |
| Current general dengue prevention practices | • Personal actions to destroy breeding sites within the home |
|  | • Personal action to reduce amount of mature mosquitoes in the home |
|  | • Awareness of actions taken by the government |
|  | • Participation in collective community action |
| Attitudes to prevention | • Methods perceived to be the most effective |
|  | • Methods perceived to be the least effective |
|  | • Reasons for not taking personal preventative action |
|  | • Satisfaction with general dengue prevention |
|  | • Perceptions of who holds responsibility for preventing dengue |
| Use of the screen | • Complete or partial coverage of windows and doors in the house |
|  | • Number of screens still hanging compared to original amount installed |
|  | • Screen cleaning and maintenance practices |
| Screen effect | • Effect on domestic mosquito amount and biting |
|  | • Effect on other pests |
| General acceptance and satisfaction with the project | • Satisfaction with the screens |
|  | • Satisfaction with installation |
|  | • Would they recommend it to another city |
|  | • Recommended areas for improvement |
