# Supplementary material for: Use and acceptance of long lasting insecticidal net screens for dengue prevention in Acapulco, Guerrero, Mexico
Source: BMC Public Health. 2014 Aug 14;14:846. doi: 10.1186/1471-2458-14-846 (PMC4152567; doi:10.1186/1471-2458-14-846)
Supplement: Supplementary file 2 — Additional file 2: Summary of the results of the satisfaction survey on the use and acceptance of long lasting insecticidal screens on doors and windows for dengue prevention in Acapulco, Guerrero, Mexico. (DOC 474 KB) [file 12889_2014_6989_MOESM2_ESM.doc]

**Additional file 2** Summary of the results of the satisfaction survey on the use and acceptance of long lasting insecticidal screens on doors and windows for dengue prevention in Acapulco, Guerrero, Mexico

|  |  |
| --- | --- |
|  |  |
|  | |
